# Supplementary material for: Loss of Glutathione-S-Transferase Theta 2 (GSTT2) Modulates the Tumor Microenvironment and Response to BCG Immunotherapy in a Murine Orthotopic Model of Bladder Cancer
Source: Int J Mol Sci. 2024 Dec 11;25(24):13296. doi: 10.3390/ijms252413296 (PMC11676541; doi:10.3390/ijms252413296)
Supplement: Supplementary file 1 [file ijms-25-13296-s001.zip › ijms-3344980-supplementary.pdf]

Supplementary Information for

Loss of Glutathione-S-transferase theta 2 (GSTT2) modulates the tumor microenvironment and response to BCG immunotherapy in a murine orthotopic model of bladder cancer

Mugdha V Patwardhan[1], Toh Qin Kane[2], Edmund Chiong[1,3,] Juwita N. Rahmat[1] and Ratha Mahendran[1*]

[1] Department of Surgery, Yong Loo Lin School of Medicine, National University of Singapore,119228, Singapore
[2] Genomics and Data Analytics Core, Cancer Science Institute of Singapore, National University of Singapore, 117599, Singapore
[3] Department of Urology, National University Hospital, National University Health System, Singapore, 119074, Singapore
* Correspondence: surrm@nus.edu.sg

*Corresponding author:

Dr Ratha Mahendran
Email: surrm@nus.edu.sg
Department of Surgery,
Yong Loo Lin School of Medicine,
National University of Singapore,
1E Kent Ridge Road,
NUHS Tower Block, Level 8,
Singapore 119228

Experimental Section

Cell culture

MB49-PSA cells were maintained in high glucose Dulbecco's Modified Eagle's Medium (DMEM) (Cytiva, MA, USA) supplemented with 10% heat-inactivated fetal bovine serum (FBS) (Biowest, Nuaille, France), 100U/mL Penicillin and 100µg/mL Streptomycin (Thermo Fisher, MA, USA) and 200ug/mL of hygromycin B (Thermo Fisher, MA, USA). Before implantation in mice, PSA expression was measured as previously described [1]. Briefly, MB49-PSA cells (1×10^6) were seeded in 24-well plates and incubated at 37°C for 24h. The supernatant was collected to assess secreted PSA by ELISA. The cells were lysed for RNA extraction and quantification of PSA expression by reverse-transcription (RT) quantitative polymerase chain reaction (RT-qPCR). MB49-PSA cells with secreted PSA > 30ng/mL per 10^6 cells and PSA expression (CT mean) = 20-25 were implanted in mice. For implantation, MB49-PSA cells were prepared in blank DMEM media.

BCG culture

BCG (Immunobladder® Intravesical, Japan BCG Laboratory, Tokyo, Japan) was plated on Middlebrook 7H10 agar ((BD DIFCO™, NJ, USA) supplemented with 0.1% Tween-80 and 10% ADC supplement (0.85% sodium chloride, 5% bovine serum albumin, 2% dextrose and 0.003% catalase, 0.05% oleic acid) [2]. Colonies were cultured in Middlebrook 7H9 media (BD DIFCO™, NJ, USA) supplemented with 0.2% glycerol (VWR Chemicals BDH®, PA, USA), 0.05% Tween-80 and 10% ADC supplement. Optical density (OD) was measured using a UV-VIS spectrophotometer (Shimadzu, Kyoto, Japan) at 600nm, and the colony forming units (CFU) were calculated using the formula: OD of 0.1 = 2.6×10^6 CFU/mL. For treatment, BCG was prepared to a final concentration of 3×10^7CFU/mL in saline (B. Braun, Penang, Malaysia). Unless stated otherwise, all reagents were from Sigma Aldrich (MO, USA).

Urine Analysis

Urinary creatinine was detected using the QuantiChrom™ Creatinine Assay Kit (BioAssay Systems, CA, USA), per manufacturer's instructions. Urine samples were diluted seven-fold in double deionized water (ddH2O). The experiment was performed in duplicate, and the absorbance was read at 510 nm using Varioskan LUX multimode microplate (Thermo Fisher, MA, USA). The creatinine concentration was calculated using the formula: Concentration (mg/dL) = Sample(Abs2 – Abs1) / Standard(Abs2 – Abs1). Urinary PSA was detected using the free-PSA ELISA kit (Abnova, Taipei, Taiwan), per manufacturer instructions; all reagents were provided within the kit. The absorbance was measured using the Varioskan LUX multimode microplate reader at 450nm. The PSA concentration was quantified against the standard curve and normalized to creatinine. Analysis was carried out on GraphPad Prism version 9 (RRID:SCR_002798).

Single-cell RNA sequencing

Bladders were mechanically disassociated by cutting into small pieces, mashing, and then digested in 2mg/mL of collagenase D (Roche, Basel, Switzerland) for 30 minutes at 37°C. The cell suspension was filtered, and RBCs were lysed, after which the cells were quantified and frozen in freezing media (10% DMSO (Sigma-Aldrich, MO, USA) in FBS) at -80°C. Cells were thawed, quantified, and 4×106 cells from each sample were fixed using the EVERCODE™ fixation kit (Parse Biosciences, WA, USA). Briefly, cells were strained and incubated sequentially with fixation solution, permeabilization solution, and neutralization buffer. Once cells were fixed, they were strained and frozen in cell buffer supplemented with 5% DMSO at -80°C.

Fixed cells were thawed, quantified, and barcoded using the Evercode™ whole transcriptome mini kit (Parse Biosciences, WA, USA). The fixed cells from each sample were diluted in dilution buffer and transferred to individual wells in a 96-well plate. Each well contained well-specific barcodes used to determine which sample the cells originated from. Intracellular RNA was reverse-transcribed to an RNA-cDNA hybrid, and the well-specific barcode was added.

The cells from each well were pooled and split again into a new 96-well plate, and a second barcode was added to each cell. This process was repeated a third time. The cells were then quantified and split into two sub-libraries. Cells were lysed to release the barcoded transcripts, which were amplified to produce double-stranded cDNA. The cDNA sub-libraries were fragmented/shortened to the appropriate size for sequencing and an Illumina adapter was ligated to the cDNA. An index PCR was carried out, during which a sub-library-specific barcode and P5 and P7 adaptors were added to the transcripts. Finally, the sample was cleaned up using Solid-phase reversible immobilization (SPRI) beads, and the cDNA quality and yield were assessed using the TapeStation (Agilent, CA, USA). The sub-libraries were sequenced with Novogene (HiSeq PE150). All reagents were provided within the kit and all procedures were carried out per manufacturer's instructions.

The Parse Biosciences split-pipe (v0.9.6p) program was used to align reads to the mouse genome (GRCm39) with Ensembl GTF annotations (Release 107) and to generate the unique molecular identifier (UMI) x cell count matrices. The filtered matrices across all samples from the two sequenced sublibraries were combined and analyzed in Seurat v4.3.0 (RRID:SCR_016341). We retained cells that pass these 3 quality control thresholds: nFeature_RNA > 300, nCount_RNA >300 and percent.mt < 10. Following quality control, UMI counts were log-normalised, and the top 2000 highly variable features were identified. To evaluate the effects of cell cycle heterogeneity, cells were assigned a cell cycle score via Seurat's CellCycleScoring function based on the expression of G2/M and S phase markers for Mus Musculus shared by the Harvard Chan School Bioinformatics Core (https://github.com/hbc/tinyatlas). To reduce the dimensionality of the dataset, we performed principal component analysis (PCA) and uniform manifold approximation and projection (UMAP). To identify the major cell clusters in the dataset, we constructed a shared nearest-neighbor graph using the first 15 PC dimensions followed by the FindClusters function with a resolution of 0.5. Cluster markers were then identified via the FindAllMarkers function using the Wilcoxon rank-sum test for differential gene expression testing,

returning only the positive biomarkers. Biomarkers with adjusted p-values less than 0.05 were retained. Clusters were manually annotated with their putative cell type identity by cross-referencing against known markers from the literature. Following cell type annotation, we identified markers that distinguish the WT and KO cells across all cells globally and within individual cell clusters. Enrichment analyses of the marker gene lists were performed against the Kyoto Encyclopedia of Genes and Genomes (KEGG) and Molecular Signatures Database (MSigDB) implemented via the R ClusterProfiler package [3]. We performed differential abundance analysis with the MiloR [4] tool to assess for changes in cellular composition across neighborhoods. The distinct [5] package was used to identify genes with statistically significant variations in expression patterns between the WT and Gstt2-KO conditions.

RNA extraction and RT-qPCR

Bladders harvested and stored in RNA-later™-ICE were weighed, and approximately 0.05g of bladder tissue was used for RNA extraction. The tissue was cut into small pieces and homogenized at 50Hz in 1mL of Trizol reagent (Thermo Fisher, MA, USA) using a tissue homogenizer (TissueLyser LT, QIAGEN, Hilden, Germany). The tissue was lysed in five 1 minute cycles, and RNA was isolated using the Trizol RNA extraction method, per the manufacturer's instructions. For DNAse treatment, 2μg of RNA was incubated with 10U/μL DNAse in 1x DNAse buffer (Roche, Basel, Switzerland), and 2U/μL of RNasin® Ribonuclease Inhibitor (Promega, WI, USA) for 5 mins. The reaction was stopped by adding 1μL of 25mM EDTA (Thermo Fisher, MA, USA) and heat inactivating at 65°C for 10 mins. RNA was then transcribed to cDNA using the High-Capacity cDNA Reverse Transcription Kit (Thermo Fisher, MA, USA), as per manufacturer's instructions. RT-qPCR was carried out in the 7500 Real-Time PCR System (Thermo Fisher, MA, USA) using the PowerUp™ SYBR™ Green Mastermix (Thermo Fisher, MA, USA) and predesigned PrimeTime® qPCR Primers from Integrated DNA Technologies (IDT) (IA, USA) (**Supplementary Table 6**. The PCR conditions were as follows: 50°C (2 minutes), 95°C (10 minutes), 95°C (15

seconds) and 60°C (1 minute). The cycle threshold (CT) maximum was set at 35 cycles, so CT values higher than 35 were taken as absence/undetected transcripts. Results were analyzed on the 7500 software. Only triplicates with a standard deviation in CT values below 0.3 were included for analysis.

Supplementary Table S1 Gene expression in the bladders of WT and KO mice

Gene	Gstt2 WT		Gstt2 KO	
	Control (n=12)	BCG-treated (n=13)	Control (n=14)	BCG-treated (n=14)
IL-6	0.948 ± 0.554	2.987 ± 5.445	1.094 ± 0.992	1.544 ± 2.254
TNF-α	1.088 ± 0.723	5.265 ± 5.995	2.254 ± 2.722	8.340 ± 7.783*
IL-1β	1.107 ± 0.728	3.296 ± 5.906	1.230 ± 1.829	2.575 ± 3.358
CD28	0.858 ± 0.478	1.255 ± 0.943	0.576 ± 0.421	1.280 ± 1.074
IL-10	1.103 ± 0.388	1.454 ± 1.885	1.920 ± 1.892	2.112 ± 1.293
PD-L1	0.996 ± 0.377	3.722 ± 4.478*	0.907 ± 0.651	2.198 ± 2.237
PD-1	0.745 ± 0.398	1.115 ± 1.384	0.723 ± 0.428	0.632 ± 0.471
CTLA-4	0.824 ± 0.490	1.177 ± 1.088	0.474 ± 561	0.504 ± 0.462
FoxP3	0.743 ± 0.457	1.256 ± 1.470	0.426 ± 0.436	0.665 ± 0.553

Data presented as Mean Relative Quantification (RQ) ± SD. Comparisons of means were performed using one-way ANOVA with Tukey's test for multiple comparisons. The mean of each group was compared to the mean of every other group. *Significant differences were observed when comparing control to BCG-treated mice (*p-value <0.05). WT – Wildtype, KO – Knockout

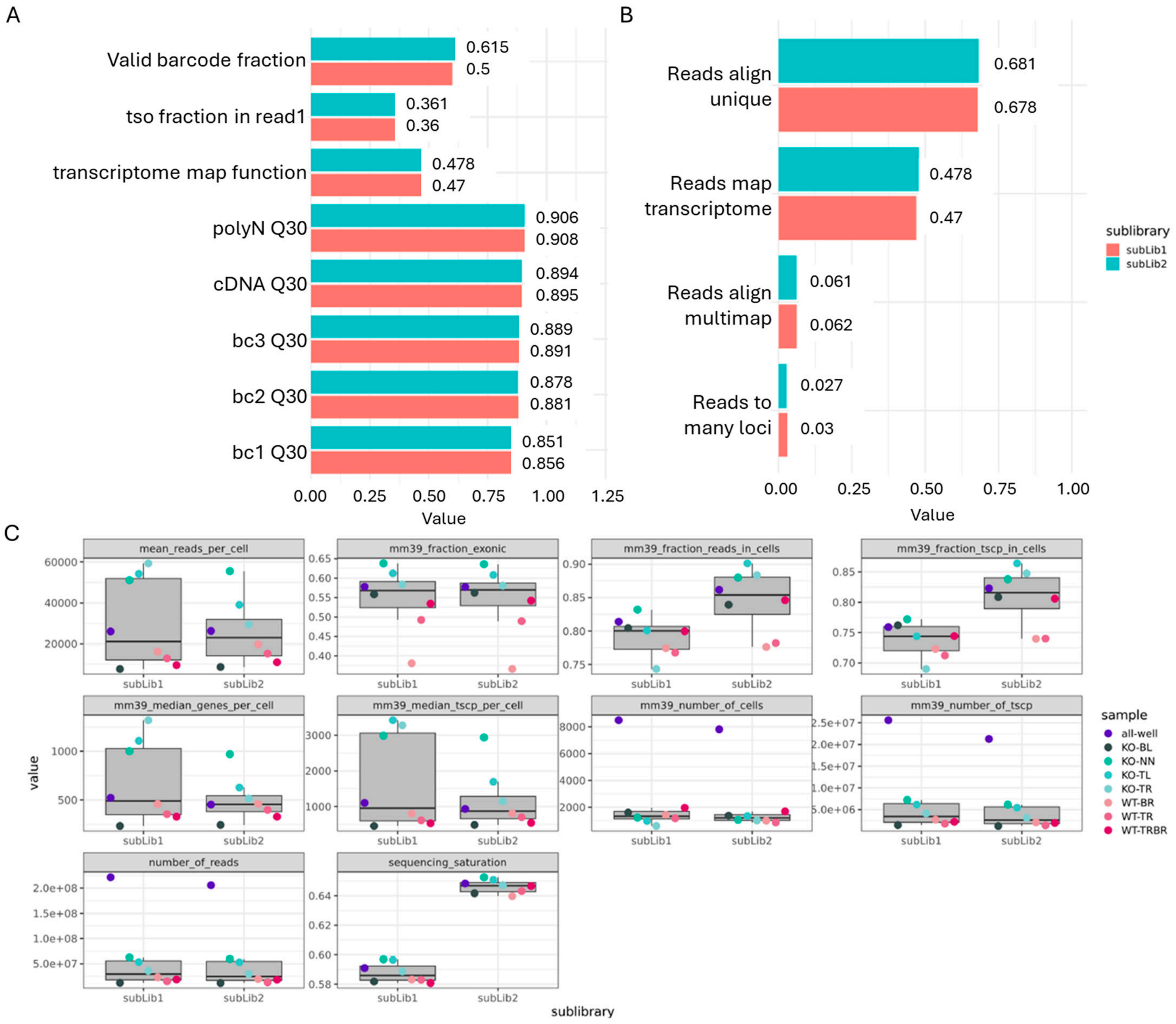
A
Valid barcode fraction
0.615
0.5
tso fraction in read1
0.361
0.36
transcriptome map function
0.478
0.47
polyN Q30
0.906
0.908
cDNA Q30
0.894
0.895
bc3 Q30
0.889
0.891
bc2 Q30
0.878
0.881
bc1 Q30
0.851
0.856
0.00 0.25 0.50 0.75 1.00 1.25
Value
B
Reads align unique
0.681
0.678
Reads map transcriptome
0.478
0.47
Reads align multimap
0.061
0.062
Reads to many loci
0.027
0.03
0.00 0.25 0.50 0.75 1.00
Value
sublibrary
subLib1
subLib2
C
mean_reads_per_cell
mm39_fraction_exonic
mm39_fraction_reads_in_cells
mm39_fraction_tscp_in_cells
mm39_median_genes_per_cell
mm39_median_tscp_per_cell
mm39_number_of_cells
mm39_number_of_tscp
number_of_reads
sequencing_saturation
value
subLib1
subLib2
sublibrary
sample
all-well
KO-BL
KO-NN
KO-TL
KO-TR
WT-BR
WT-TR
WT-TRBR

Supplementary Figure S1. Quality control analysis of single-cell RNA sequencing output. WT and KO C57BL6/J female mice were implanted orthotopically with MB49-PSA BC cells and one-week post implantation, the mice were treated with weekly intravesical BCG instillations for 4 weeks. One day after the fourth instillation, the bladders were harvested, and single cells were isolated for RNA sequencing. Single-cells were barcoded using the Evercode whole-transcriptome kit from Parse Biosciences (WA, USA) and sequenced. The data output was aligned to the mouse genome (GRCm39) with the split-pipe (v0.9.6p) program from Parse Biosciences. (A) The quality (Phred quality scores, valid barcodes, polyN fraction), (B) alignment statistics and (C) sequencing depth (segregated by sample), were assessed across both sub-libraries.

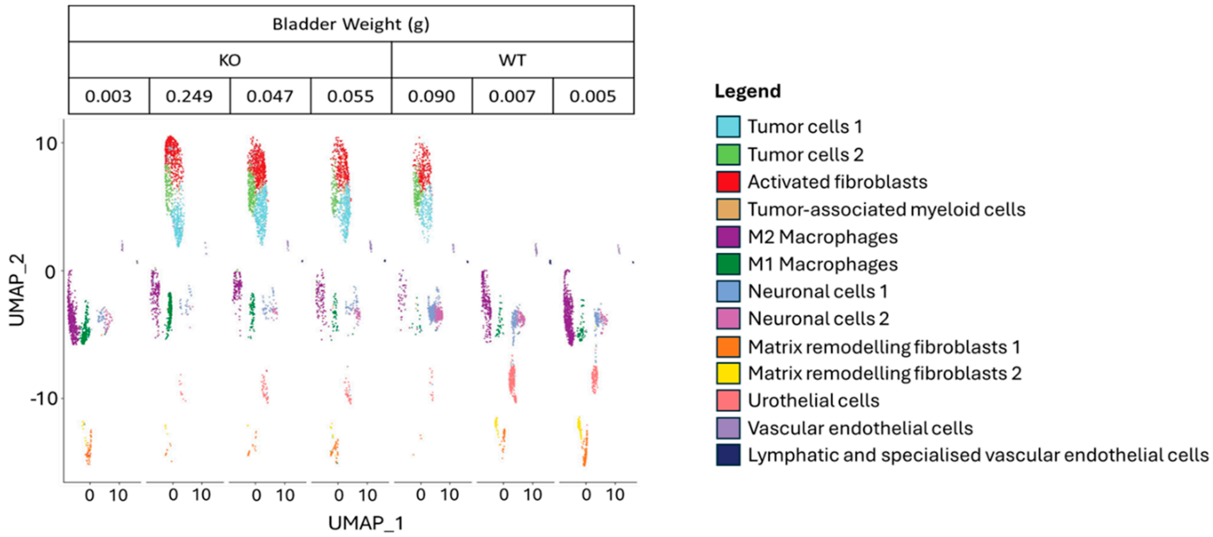

Supplementary Figure S2. Single-cell RNA sequencing of whole bladders from WT and KO mice. MB49-PSA BC cells were implanted orthotopically into WT and KO C57 BL6/J female mice. The mice were treated with weekly BCG instillations one week post-implantation for 4 weeks. Bladders were harvested one day after the fourth BCG instillation, and single cells were isolated for single-cell RNA sequencing. Standard principal component analysis (PCA) and uniform manifold approximation and projection (UMAP) were used to cluster cells, and the top differentially expressed genes between the clusters were used to determine cell type. The UMAP plots were segregated by sample and correlated with post-harvest bladder weights.

Supplementary Table S2. Top 10 differentially expressed genes between WT and KO backgrounds within each cell type

Cell Cluster	Upregulated in WT		Upregulated in KO	
	Gene	Log2FC	Gene	Log2FC
Urothelial cells	Lrmda	1.60	Gm19951	1.86
	Ankfn1	1.47	Rpph1	1.81
	Trp63	1.33	Cdk8	1.62
	Fmo5	1.26	Mki67	1.38
	Ugt1a6a	1.22	Gm48099	1.26
	Sh3gl2	1.10	Tmsb10	1.21
	Atrn	0.84	Hmga2	1.08
	Mecom	0.84	Vim	1.06
	Malat1	0.59	Ctsl	1.06
			Ccbe1	1.02
Tumor cells 1	Jund	1.40	Gm19951	2.28
	Cdk8	1.26	Gphn	1.53
	Rplp2	1.22	Cmss1	1.38
	Wdr89	1.20	Gm30835	1.13
	S100a6	1.17	Ahnak	1.09
	Rplp1	1.16	Filip1l	1.02
	Rps21	1.09	Mki67	0.98
	Rps12	0.99	Plec	0.78
	Rps9	0.95	Rn18s-rs5	0.77
	Rpl41	0.90	Ybx1	0.73
Tumor cells 2	Cdk8	1.46	Gm19951	2.51
	Jund	1.40	Gphn	1.81
	Rps21	1.17	Peak1	1.69
	Psmd13	1.12	Cmss1	1.58
	Dusp6	1.04	Filip1l	1.30
	Cct8	1.00	Gm30835	1.25
	Wdr89	1.00	Rn18s-rs5	1.20
	Rplp2	0.92	Ahnak	0.80
	Ccnb1ip1	0.88	mt-Nd2	0.78
	Rplp1	0.85	mt-Co1	0.71
Activated fibroblasts	Cdk8	1.38	Gm19951	2.22
	Jund	1.34	Gphn	1.44
	Wdr89	1.23	Cmss1	1.40
	Rplp2	1.22	Gm30835	1.32
	S100a6	1.10	Ahnak	1.29
	Ier3	1.07	Filip1l	1.23
	Rps12	1.02	Peak1	1.21
	Rplp1	1.01	Rps2	0.94
	Rps21	0.95	Nolc1	0.81
	Gm10076	0.93	Rn18s-rs5	0.75
Matrix remodelling fibroblasts 1	Sntg1	1.78	Cacna1c	2.45
	Cd55	1.62	Rpph1	2.40
	Cxcl10	1.59	Nrg1	2.16
	Olfr1033	1.56	Peak1	1.97
	Egr1	1.55	Plec	1.91

	Celf2	1.54	Myh9	1.83
	Ccl2	1.53	Tnc	1.75
	B2m	1.53	Piezo2	1.61
	Saa3	1.53	Col12a1	1.57
	Meg3	1.49	Cxcl14	1.50
Matrix remodelling fibroblasts 2			Rpph1	2.17
			Prpf38b	1.57
			Plec	1.52
			Adam12	1.34
			Cdk8	1.26
			Anln	1.24
			Plat	1.10
			Nav3	1.03
			Golim4	0.98
			Ppl	0.91
M1 Macrophages	Gm10800	1.42	Peak1	1.49
	Cxcl10	1.36	Gphn	1.36
	Fmo5	1.22	Alcam	1.25
	Aff1	1.12	Baz2b	1.14
	Irf1	0.98	Slfn4	1.12
	Dennd4a	0.97	Myh9	0.98
			Lilrb4b	0.88
			Cmss1	0.61
			Rn18s-rs5	0.59
			Lars2	0.42
M2 Macrophages	Saa3	1.76	Peak1	2.23
	Abi1	1.43	Rpph1	1.99
	Ccl5	1.37	Gphn	0.97
	Hdac9	1.30	Plec	0.93
	Ly6a	1.30	Cdk8	0.84
	Slc7a2	1.25	Abcg3	0.83
	Fth1	1.19	Lars2	0.76
	Kdm6b	1.14	Gm5150	0.71
	C3	1.13	Myh9	0.69
	Dock10	1.12	Tgfbi	0.68
Tumor-associated myeloid cells	Tinag	1.53		
	Otoa	0.93		
Neurons 1	Fgfr2	0.94	Gm19951	1.85
			Rpph1	1.65
			Gphn	1.33
			Ahnak	1.30
			Rn18s-rs5	1.30
			Cmss1	1.27
			Peak1	1.24
			Eif3a	1.21
			Ybx1	1.17
			Serbp1	1.16
Neurons 2	Gm10800	0.60	Gm19951	1.71
			Cxcl2	1.42

		Rn18s-rs5	1.40
		Cmss1	1.34
		Rpl23	1.19
		Rpph1	1.18
		Neat1	1.16
		Gbp2	1.10
		Fth1	1.08
		Ftl1	1.05
Vascular endothelial cells		Col4a1	1.43
		Col4a2	1.36
		Plec	1.30
		Cdk8	0.63

Supplementary Table S3. Differentially expressed genes between WT and KO backgrounds observed across multiple cell types

Upregulated in	Gene	Log2 (Fold Change)	Function
WT	Mecom	1.91	Stem Cell, Akt mTOR pathway
	Sh3gl2	1.71	Tumor suppressor, intracellular signalling
	Fmo5	1.67	NRF2 activation,
KO	Peak1	1.72	Cell Migration/Metastasis
	Rpph1	1.67	STAT3 activation, Inflammation
	Gphn	1.52	Anchoring protein, mTOR signalling
	Plec	1.28	Migration/Metastasis
	Ahnak	1.24	Bladder cancer proliferation, Immune infiltration
	Cmss1	1.08	Cancer Marker, Immune infiltration
	Hmga2	0.79	Proliferation, EMT, Activation of NF-kB, MAPK and Akt-mTOR pathways

WT – Wildtype, KO – Knockout

Supplementary Table S4. Top differentially expressed genes between WT and KO backgrounds

Upregulated in	Gene	Log2 (Fold Change)	Function
WT	Saa3	2.20	Inflammation, Immune Infiltration, CAF
	Gm10800	1.93	-
	Mecom	1.91	Stem Cell, Akt mTOR pathway
	Ankfn1	1.88	Cell polarity, Cancer marker
	C3	1.82	Complement immune response, immune infiltration, T-cell exhaustion
	Sh3gl2	1.71	Tumor suppressor, intracellular signalling
	Fmo5	1.67	NRF2 activation,
	Tshz2	1.64	Tumor promoter/suppressor
	Rora	1.61	Regulation of type II immune cells, expressed in BC
	Prkn	1.56	Survival, inhibition of mitochondrial ROS
	Gsta4	1.27	Detoxification
KO	Peak1	1.72	Cell Migration/Metastasis
	Rpph1	1.67	STAT3 activation, Inflammation
	Gm19951	1.63	-
	Gphn	1.52	Anchoring protein, mTOR signalling
	Plec	1.28	Migration/Metastasis
	Ahnak	1.24	BC proliferation, Immune infiltration
	S100a9	1.20	TLR4 expression, Ca2+ signalling, Immune infiltration
	Mki67	1.10	Proliferation
	Slfn4	1.10	Macrophage activation, type I IFN target gene,
	Cmss1	1.08	Cancer Marker, Immune infiltration
	Hmga2	0.79	Proliferation, EMT, Activation of NF-kB, MAPK and Akt-mTOR pathways
	Slc2a1	0.53	Glucose Transporter, metabolism

(independent of cell type)

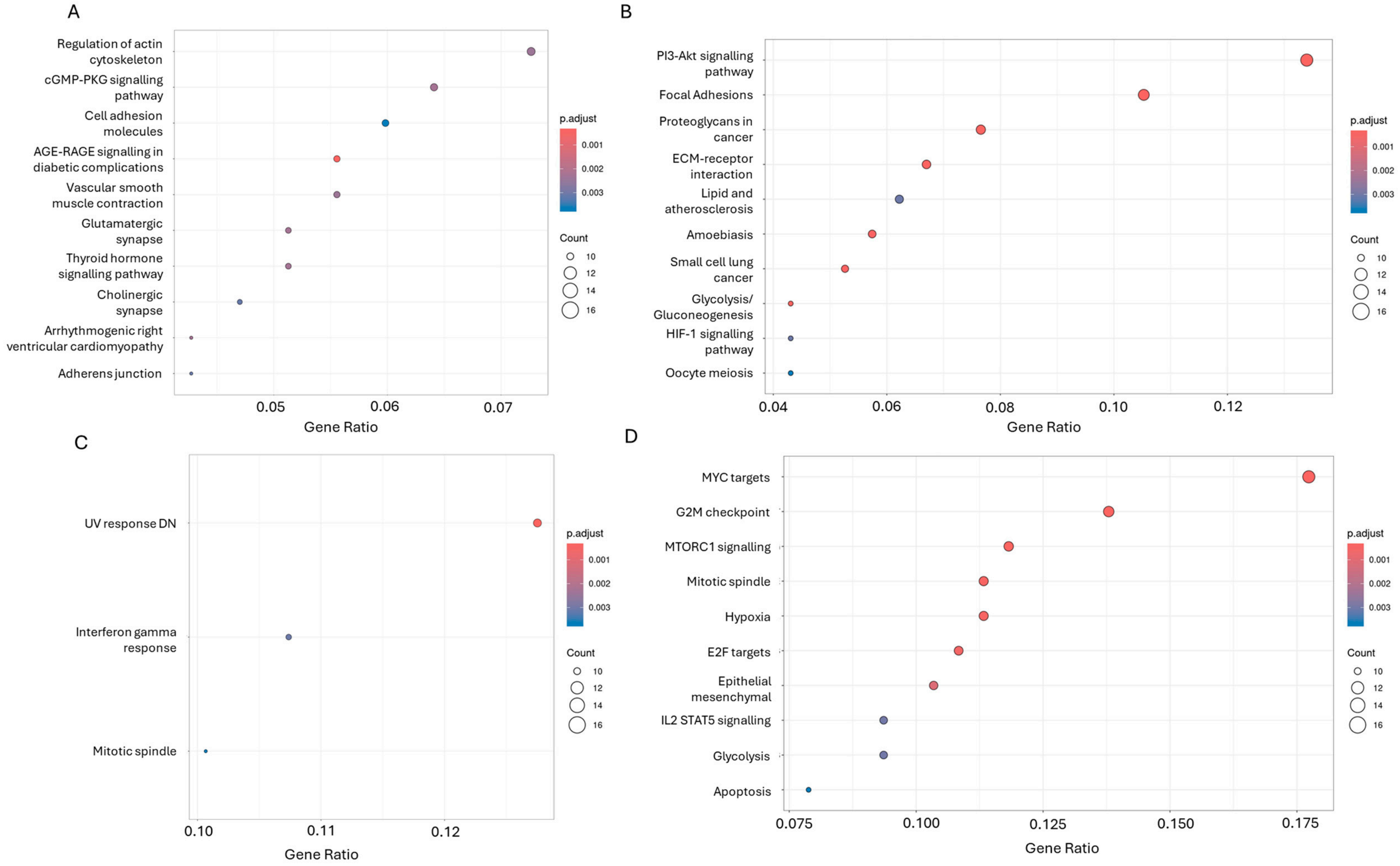

Supplementary Figure S3. Comparison of enriched pathways between WT and KO background. The “FindAllMarkers” function was used to identify differentially expressed genes (DEG) between WT and KO backgrounds and the top DEGs were used to perform enrichment analysis against the Kyoto Encyclopedia of Genes and Genomes (KEGG) database in (A) WT and (B) KO mice. The Molecular Signatures Database (MSigDB) was used to idenfity enriched pathways in (C) WT and (D) KO mice.

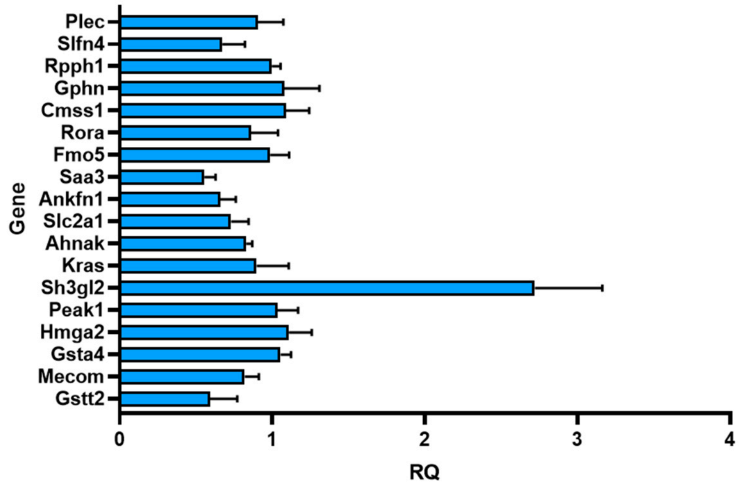

Supplementary Figure S4. Gene expression in MB49-PSA cells. MB49-PSA cells were stimulated in vitro with BCG for 24h, and RNA was extracted for analysis. Gene expression was assessed through quantitative real-time PCR (RT-qPCR). The expression of each gene in BCG-stimulated MB49-PSA cells was normalized to Ribosomal Protein S27a (RPS27a), an endogenous control, and quantified relative to untreated MB49-PSA cells. Data is presented as relative quantification (RQ); error bars represent standard deviation (n=3).

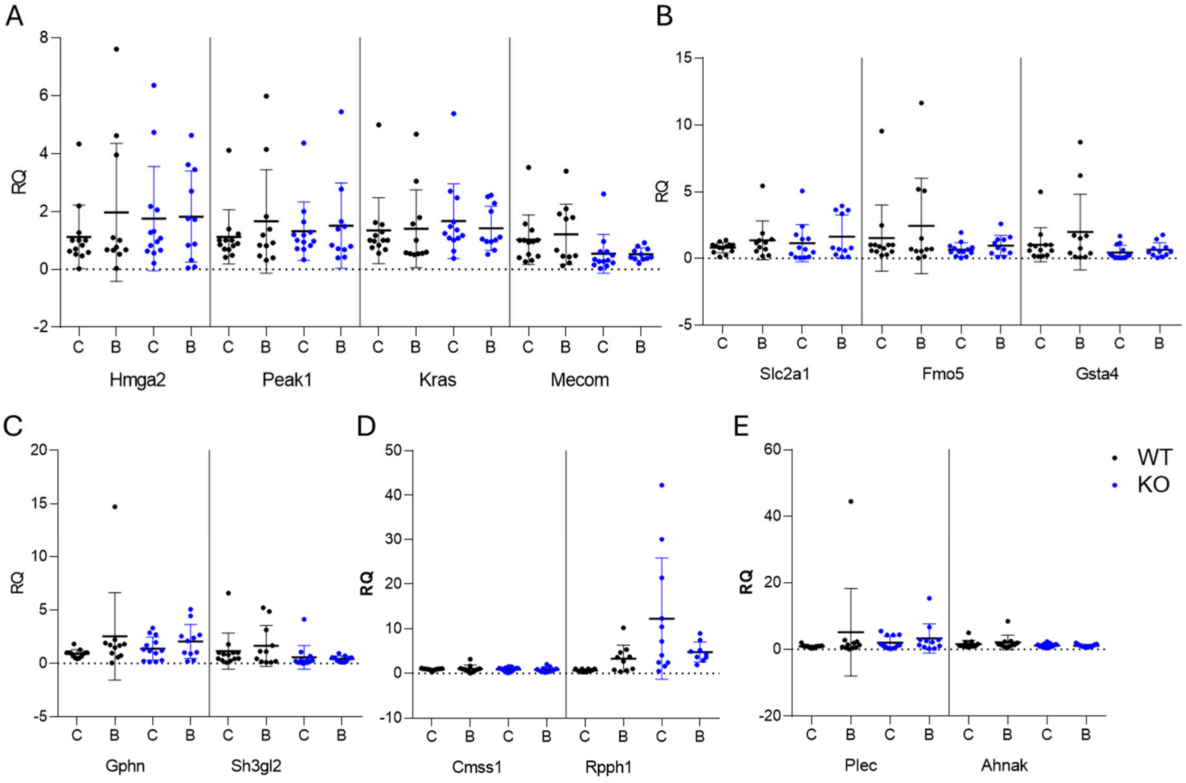

Supplementary Figure S5: Gene expression in WT and KO bladders. MB49-PSA bladder cancer cells were implanted orthotopically into Gstt2 WT and KO C57BL6/J female mice. The mice were treated with weekly BCG instillations one week post-implantation for 4 weeks. Bladders were harvested one day after the fourth BCG instillation and RNA was extracted for quantitative real-time PCR. (A) Expression of genes involved in tumorigenic (B) metabolic (C) neuronal (D) immune and (E) structural pathways was compared in WT and KO mice. Comparisons of means were performed using one-way ANOVA and the mean of each group was compared to the mean of every other group using Tukey's test for multiple comparisons. There were no significant differences. WT – Wildtype, KO – Knockout

Supplementary Table S5. Combinations of select genes and their impact on clinical outcomes in BC patients

Number of genes	Genes	Hazard Ratio (HR)* (Logrank p-value, HR p-value)		
		Total	Papillary	Non-papillary
**Overall survival**				
1	Saa3p	0.84 (0.25, 0.26)	0.98 (0.94, 0.94)	0.82 (0.27, 0.27)
	Rora	1.0 (0.91, 0.91)	0.83 (0.55, 0.55)	1.0 (0.91, 0.92)
	Ankfn1	0.86 (0.32, 0.32)	0.88 (0.69, 0.70)	0.94 (0.74, 0.74)
	Gstt2	1.2 (0.23, 0.23)	0.79 (0.46, 0.47)	1.4 (0.045, 0.046)
	Rpph1	0.9 (0.49, 0.49)	0.98 (0.94, 0.94)	0.88 (0.47, 0.47)
	Plec	1.2 (0.31, 0.31)	0.97 (0.91, 0.92)	1.3 (0.17, 0.17)
	Gphn	0.99 (0.93, 0.93)	1.2 (0.59, 0.59)	1 (0.86, 0.85)
	Fmo5	0.81 (0.16, 0.16)	0.96 (0.9, 0.9)	0.89 (0.53, 0.53)
	Cmss1	1.2 (0.22, 0.22)	2.3 (0.012, 0.015)	1 (0.89, 0.87)
	Ahnak	1.6 (0.0022, 0.0024)	1.4 (0.27, 0.27)	1.5 (0.028, 0.029)
	Sh3gl2	0.83 (0.23, 0.23)	1.2 (0.62, 0.61)	0.88 (0.47, 0.48)
	Mecom	0.8 (0.15, 0.15)	0.68 (0.23, 0.24)	0.94 (0.74, 0.73)
	Slc2a1	1.2 (0.18, 0.18)	0.69 (0.25, 0.26)	1.6 (0.0097, 0.01)
	Gsta4	1.3 (0.14, 0.14)	1.3 (0.48, 0.48)	1.3 (0.11, 0.11)
	Kras	1.0 (0.91, 0.91)	1.5 (0.18, 0.18)	0.91 (0.61, 0.61)
	Peak1	1.1 (0.67, 0.67)	1.1 (0.83, 0.82)	1.1 (0.71, 0.71)
	Hmga2	1.3 (0.11, 0.11)	1.7 (0.08, 0.083)	1 (0.94, 0.93)
10	Hmga2, Peak 1, Kras, Gsta4, Slc2a1, Ahnak, Cmss1, Gphn, Plec, Gstt2	1.3 (0.13, 0.13)	1 (0.93, 0.92)	1.3 (0.09, 0.09)
12	Hmga2, Peak 1, Kras, Gsta4, Slc2a1, Mecom, Ahnak, Cmss1, Fmo5, Gphn, Plec, Gstt2	1.1 (0.58, 0.57)	1.2 (0.49, 0.49)	1.1 (0.65, 0.66)
13	Hmga2, Peak 1, Kras, Gsta4, Slc2a1, Mecom,	0.97 (0.85, 0.85)	1.1 (0.77, 0.77)	1.1 (0.74, 0.74)

	Ahnak, Cmss1, Fmo5, Gphn, Plec, Gstt2, Ankfn1			
2	Gstt2, Ahnak	1.3 (0.071, 0.071)	0.98 (0.98, 0.96)	1.5 (0.025, 0.025)
3	Gstt2, Ahnak, Gphn	1.1 (0.49, 0.49)	0.93 (0.82, 0.83)	1.2 (0.4, 0.4)
**Disease-free survival**				
1	Saa3p	1.5 (0.018, 0.019)	1.7 (0.13, 0.14)	1.3 (0.18, 0.18)
	Rora	1.1 (0.58, 0.58)	1.1 (0.78, 0.78)	1.0 (0.89, 0.88)
	Ankfn1	1.2 (0.21, 0.22)	1.3 (0.52, 0.52)	1.3 (0.14, 0.15)
	Gstt2	1.6 (0.009, 0.0096)	1.2 (0.6, 0.6)	1.8 (0.0022, 0.0025)
	Rpph1	1.5 (0.018, 0.02)	1.6 (0.21, 0.21)	1.5 (0.035, 0.037)
	Plec	1.4 (0.036, 0.037)	1.3 (0.47, 0.47)	1.5 (0.04, 0.041)
	Gphn	1.4 (0.075, 0.076)	1.5 (0.22, 0.22)	1.4 (0.11, 0.11)
	Fmo5	1.3 (0.13, 0.13)	1.5 (0.23, 0.23)	1.4 (0.078, 0.081)
	Cmss1	1.6 (0.0089, 0.0095)	1.7 (0.13, 0.14)	1.5 (0.055, 0.055)
	Ahnak	1.6 (0.011, 0.011)	1.1 (0.88, 0.88)	1.7 (0.0087, 0.0091)
	Sh3gl2	1.1 (0.63, 0.63)	1.2 (0.56, 0.56)	1.2 (0.28, 0.28)
	Mecom	1.2 (0.31, 0.31)	1.6 (0.21, 0.21)	1.2 (0.38, 0.39)
	Slc2a1	1.5 (0.019, 0.019)	1.4 (0.35, 0.35)	1.5 (0.048, 0.048)
	Gsta4	1.4 (0.075, 0.075)	2.0 (0.052, 0.058)	1.2 (0.41, 0.41)
	Kras	1.4 (0.051, 0.052)	2.0 (0.057, 0.062)	1.3 (0.21, 0.21)
	Peak1	1.5 (0.023, 0.024)	2.0 (0.058, 0.064)	1.4 (0.07, 0.072)
	Hmga2	1.2 (0.31, 0.31)	1.7 (0.13, 0.14)	0.95 (0.77, 0.78)
12	Hmga2, Peak 1, Kras, Gsta4, Slc2a1, Mecom, Ahnak, Cmss1, Fmo5, Gphn, Plec, Gstt2	1.8 (0.00066, 0.00079)	2.8 (0.0046, 0.0068)	1.6 (0.022, 0.023)
13	Hmga2, Peak 1, Kras, Gsta4, Slc2a1, Mecom, Ahnak, Cmss1, Fmo5,	1.7 (0.0024, 0.0027)	2 (0.0045, 0.05)	1.7 (0.01, 0.011)

	Gphn, Plec, Gstt2, Ankfn1			
11	Hmga2, Peak 1, Kras, Slc2a1, Ahnak, Cmss1, Fmo5, Gphn, Plec, Gstt2, Ankfn1	2 (0.000068, 0.000093)	3.1 (0.0019, 0.0031)	1.8 (0.0042, 0.0047)
2	Gstt2, Ahnak	1.5 (0.026, 0.026)	0.99 (0.98, 0.98)	1.7 (0.0088, 0.0093)
4	Gsst2, Ahnak, Kras, Peak1	1.4 (0.044, 0.045)	1.7 (0.13, 0.14)	1.3 (0.14, 0.14)
5	Gsst2, Ahnak, Kras, Peak1, Plec	1.6 (0.0042, 0.0046)	1.7 (0.15, 0.16)	1.6 (0.015, 0.016)

*Normalised to Rps27a

Supplementary Table S6. Predesigned primers for RT-qPCR

**Probe**	**Catalogue/ID**
RPs27a	Mm.PT.58.5998105.g
IL-6	Mm.PT.58.10005566
TNF-α	Mm.PT.58.12575861
IL-1β	Mm.PT.58.41616450
IL-10	Mm.PT.58.13531087
CD28	Mm.PT.58.12148064
PD-L1	Mm.PT.58.43032118
PD-1	Mm.PT.58.29141957
CTLA-4	Mm.PT.58.30832695
Foxp3	Mm.PT.58.31735290
Hmga2	Mm.PT.58.6270036
Peak1	Mm.PT.58.32569499
Slc2a1	Mm.PT.58.7590689
Kras	Mm.PT.58.28643382
Ahnak	Mm.PT.56a.13518996
Gsta4	Mm.PT.58.1703086
Mecom	Mm.PT.58.14269987
Sh3gl2	Mm.PT.58.30678897
Saa3	Mm.PT.58.10949783
Ankfn1	Mm.PT.58.30297252
Fmo5	Mm.PT.58.10533765
Rora	Mm.PT.58.32675621
Rpph1	Mm.PT.58.42355525.g
Plec	Mm.PT.58.17372476
Gphn	Mm.PT.58.12477923
Slfn4	Mm.PT.58.8668504
Cmss1	Mm.PT.56a.29065444

References

1. Tham, S.M.; Esuvaranathan, K.; Mahendran, R. A Murine Orthotopic Bladder Tumor Model and Tumor Detection System. *J Vis Exp* **2017**, doi:10.3791/55078.
2. Rahmat, J.N.; Esuvaranathan, K.; Mahendran, R. Bacillus Calmette-Guerin induces cellular reactive oxygen species and lipid peroxidation in cancer cells. *Urology* **2012**, *79*, 1411 e1415-1420, doi:10.1016/j.urology.2012.01.017.
3. Wu, T.; Hu, E.; Xu, S.; Chen, M.; Guo, P.; Dai, Z.; Feng, T.; Zhou, L.; Tang, W.; Zhan, L.; et al. clusterProfiler 4.0: A universal enrichment tool for interpreting omics data. *Innovation (Camb)* **2021**, *2*, 100141, doi:10.1016/j.xinn.2021.100141.
4. Dann, E.; Henderson, N.C.; Teichmann, S.A.; Morgan, M.D.; Marioni, J.C. Differential abundance testing on single-cell data using k-nearest neighbor graphs. *Nat Biotechnol* **2022**, *40*, 245-253, doi:10.1038/s41587-021-01033-z.
5. Tiberi, S.; Crowell, H.L.; Samartsidis, P.; Weber, L.M.; Robinson, M.D. distinct: a novel approach to differential distribution analyses. **2022**, doi:10.1101/2020.11.24.394213.
